# A Machine Learning Approach for Knee Injury Detection from Magnetic Resonance Imaging

**DOI:** 10.3390/ijerph20126059

**Published:** 2023-06-06

**Authors:** Massimiliano Mangone, Anxhelo Diko, Luca Giuliani, Francesco Agostini, Marco Paoloni, Andrea Bernetti, Gabriele Santilli, Marco Conti, Alessio Savina, Giovanni Iudicelli, Carlo Ottonello, Valter Santilli

**Affiliations:** 1 Department of Anatomical and Histological Sciences, Legal Medicine and Orthopedics, Sapienza University, 00185 Rome, Italy; anxhelo.diko@uniroma1.it (A.D.); francescoagostini.ff@gmail.com (F.A.); marco.paoloni@uniroma1.it (M.P.); andrea.bernetti@uniroma1.it (A.B.); gabriele.santilli@uniroma1.it (G.S.); ma.conti@uniroma1.it (M.C.); alessio.savina@uniroma1.it (A.S.); giovanni.iudicelli@uniroma1.it (G.I.); valter.santilli@uniroma1.it (V.S.); 2 Department of Computer Science Sapienza, University of Rome, 00198 Rome, Italy; 3 San Salvatore Hospital, Department of Biotechnological and Applied Clinical Sciences, University of L’Aquila, Vetoio Stree, 67100 L’Aquila, Italy; lucagiuliani92@virgilio.it; 4 Fisiocard Medical Centre, Via Francesco Tovaglieri 17, 00155 Rome, Italy; carlo.ottonello@gmail.com

**Keywords:** knee MRI, machine learning, MRI, radiology, automated analysis

## Abstract

The knee is an essential part of our body, and identifying its injuries is crucial since it can significantly affect quality of life. To date, the preferred way of evaluating knee injuries is through magnetic resonance imaging (MRI), which is an effective imaging technique that accurately identifies injuries. The issue with this method is that the high amount of detail that comes with MRIs is challenging to interpret and time consuming for radiologists to analyze. The issue becomes even more concerning when radiologists are required to analyze a significant number of MRIs in a short period. For this purpose, automated tools may become helpful to radiologists assisting them in the evaluation of these images. Machine learning methods, in being able to extract meaningful information from data, such as images or any other type of data, are promising for modeling the complex patterns of knee MRI and relating it to its interpretation. In this study, using a real-life imaging protocol, a machine-learning model based on convolutional neural networks used for detecting medial meniscus tears, bone marrow edema, and general abnormalities on knee MRI exams is presented. Furthermore, the model’s effectiveness in terms of accuracy, sensitivity, and specificity is evaluated. Based on this evaluation protocol, the explored models reach a maximum accuracy of 83.7%, a maximum sensitivity of 82.2%, and a maximum specificity of 87.99% for meniscus tears. For bone marrow edema, a maximum accuracy of 81.3%, a maximum sensitivity of 93.3%, and a maximum specificity of 78.6% is reached. Finally, for general abnormalities, the explored models reach 83.7%, 90.0% and 84.2% of maximum accuracy, sensitivity and specificity, respectively.

## 1. Introduction

The knee is a complex joint crucial in supporting the body and allowing for a range of movements essential for daily living activities. Unfortunately, knee injuries are common and can significantly impact a person’s quality of life [1,2]. One of the most frequently diagnosed knee injuries is meniscus tears, which can result from traumatic injury or degenerative changes due to age or overuse and cause ongoing knee pain, swelling, and stiffness, leading to a decline in functionality [3,4]. According to the literature, the incidence of meniscus tears is estimated to be 60–70 per 100,000 individuals per year, with a higher incidence among males in all age groups, ranging from 2.5:1 to 4:1 [5]. Moreover, in the United States, meniscus tears are the most frequent intra-articular knee injury [6]. They are the leading cause of orthopedic surgical procedures. Another common knee abnormality is bone marrow edema (BME), which causes ongoing pain and affects medial-aged men and young women, with a higher incidence in men. BME is often a migratory phenomenon and can occur bilaterally in over 40% of patients [7,8,9]. Based on the crucial role of knee structure on people’s functional activity, both medial meniscus and BME, and other symptomatic knee pathologies in general, can significantly impact a person’s quality of life in terms of mobility. For this reason, identifying knee injuries as early as possible is crucial to prevent their progression into severe conditions. Due to its high accuracy, magnetic resonance imaging (MRI) is currently the gold standard for assessing knee disorders, including meniscus tears, BME, and other injuries. Furthermore, the negative predictive value of Knee MRI is nearly 100% for structures such as meniscus tears and BME, and very high for traumatic meniscal and cruciate tears. Based on this property of high negative predictive value, MRI it can serve as a non-invasive method to filter out healthy patients who do not need surgical interventions with high accuracy [10]. However, MRIs come with much detail, making their interpretation time consuming and prone to errors, even among experienced radiologists. The issue becomes even more concerning when radiologists have to deal with a huge number of MRIs in a short period. In this regard, according to Kim and Mansfield in their retrospective study [11], radiologists tend to make errors in 30% of cases when evaluating MRIs, and 66% of these errors are of a musculoskeletal nature. To this end, as the field of radiology advances and demands for precision increase, there is a growing need for automation to improve efficiency and reduce errors in evaluating injuries.

To serve such a purpose, machine learning algorithms recently emerged as a promising discipline that can help build automated tools to assist radiologists in their activity [12]. Machine learning is a multidisciplinary field used in many application domains, such as computer vision [13], natural language processing [14], and medical domains [15], that converts images [10] into structured and semi-structured data [15], using algorithms that automatically improve from experience. It is often considered a subfield of artificial intelligence, which builds mathematical models and learns generalization patterns from data, which are then used to make predictions on unobserved instances [16,17]. Based on these properties of machine learning, many works in the literature addressed the use of such algorithms for medical imaging in many applications, such as skin cancer classification [18], diabetic retinography [19], lung noodle detection [20], etc. Besides these applications, machine learning models made their way into knee MRI imaging. In [10], the authors propose a method based on learning algorithms to diagnose several knee injuries using MRIs. Specifically, they rely on convolutional neural networks [21] (CNN), which are machine learning algorithms used to analyze and extract information from images, to model and extract meaningful patterns from knee MRIs that can relate to the type of injury. Additionally, in [22], three-dimensional CNNs were developed to detect the regions of interest within MRI studies and grade abnormalities in the cartilage, bone marrow, menisci, and anterior cruciate ligament (ACL). Inspired by these works, this study proposes a machine learning algorithm capable of using learning to identify knee pathologies from MRIs. The pathologies considered are medial meniscus tear, BME, and general abnormality (any kind of lesion or fracture out of those present in the data used for the study) of the knee. In the proposed approach, in a departure from other works, a feature fusion pre-processing step is employed in order to enhance the level of information provided via each MRI and ease the learning process of the model. Additionally, a series of experiments are conducted on a private cohort of 564 patients to evaluate the model capability as an automated tool in terms of accuracy, specificity, and sensitivity. It is worth mentioning that to the best of our knowledge, this is the first work in literature that uses a real-life imaging protocol for MRI, meaning that the dataset contains multiple types of MRI images per examination according to the evaluation protocols used by radiologists at the specific institute from which the data are obtained. Finally, the possibility of such tools assisting radiologists in a clinical setting based on the produced results is discussed.

To summarize, the main contributions of this study are as following:

1. A machine learning model based on CNNs used to classify knee injuries from MRI is proposed;
2. To the best of our knowledge, this is the first work to propose the use of a pre-processing phase to extend the channels of each MRI slice and create an enhanced version of the image useful for identifying knee injuries;
3. Extensive experiments are conducted on a private dataset obtained following real-life imaging protocols, achieving remarkable results.

## 2. Materials and Methods

### 2.1. Data Pre-Processing

The original MRI series were stored in Digital Imaging and Communication in Medicine (DICOM) [23] with shapes s×h×w, where s is the number of slices in the DICOM series, while h and w are the height and width of each image slice in the spatial dimension. To have uniform shapes (required by machine learning models), each image was reshaped to s×224×224 using Python programming language version 3.8 (Python Software Foundation, Wilmington, DE, USA) and libraries such as pydicom and Monai.

One common issue that arises when working with images is the varying pixel intensity. In order to deal with this issue, a pixel normalization policy was adopted. Firstly, with the help of 4 expert clinicians, an intensity range of interest for each knee injury present in this work was identified, and all of the pixels with values outside the intensity range were set to 0. Next, the mean pixel intensity and standard deviation from the entire training set was extracted and used to normalize the pixels of each MRI series according to:

(1) $$z=\frac{x-\overset{-}{x}}{\sigma }$$

where z is the normalized value, x represents the unnormalized pixels, $\overset{-}{x}$ is the mean pixel intensity value, and σ is the standard deviation of the pixel intensity.

**Figure 1:**
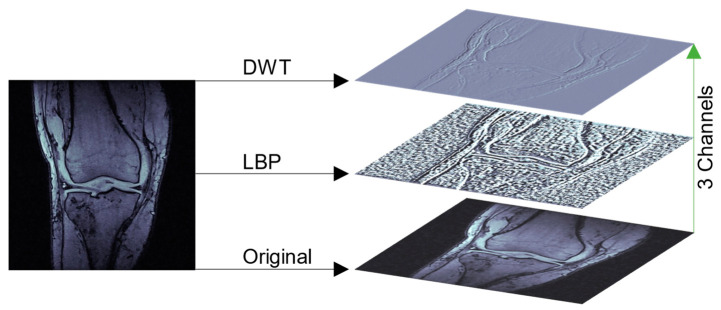
Illustration of channel formation. As can be seen in original slice, two different algorithms are applied, i.e., the DWT and LBP, the results of which are used to create a three-channel representation of the original grayscale image.

MRI images usually come in a grayscale format, meaning that each slice had one color channel representing the gray intensity that defined the details regarding the different structures comprising the knee. Inspired by [24], this work used a feature fusion mechanism that further combined three different representations of each MRI slice along the color channel dimension to enhance the amount of information. Specifically, local binary pattern [25] (LBP) and discrete wavelet transform [26] (DWT) were used to create two alternative representations of each slice, introducing a wide variety of features that characterize different knee structures. Afterward, these representations, together with the original slice, were stacked together to form an image of shape s×3×224×224, where 3 is the number of channels. The operation is illustrated in Figure 1.

### 2.2. Proposed Method

**Figure 2:**
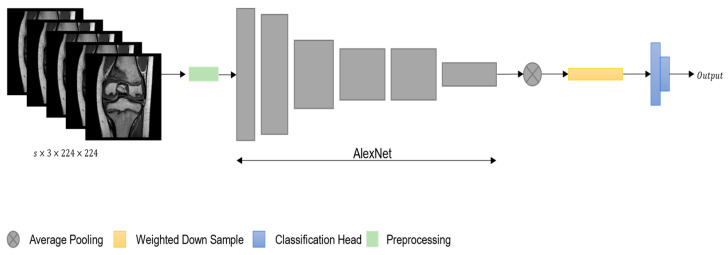
Illustration of single stream KNet architecture.

MRIs are represented as sequences where each element can be seen as a 2D image that contains a set of intensities characterizing different structures of the knee. Like normal images, the set of intensities contains important structural and local information that is essential for modeling the different patterns that can identify injuries present in different parts of the knee. To be able to extract such information, according to the literature, CNN-based algorithms are often used. These algorithms are designed to extract local and global information for images that serve a given task [21]. We noted that this property of CNNs could be used to model the patterns of the knee present in MRIs. Indeed, the model proposed in this work, which was named KNet, was based on CNNs. Specifically inspired by MRNet [10], it employed a version of CNN, which was named AlexNet [27], as the main building block used to extract the features that characterize the knee from MRI, as illustrated in Figure 2. To better understand the working cycle of the model, we supposed that an input MRI of shape s×3×224×224 was given to the networks. Firstly, the network iterated over the input along the slice dimension to extract features at the slice level using AlexNet and produce feature vectors of shape s×256×7×7 for each slice. To proceed, the model used an average pooling function that first downsampled the feature vector of each slice into a global feature vector at slice level of dimensionality s×256, and then a linear transformation was used to produce a weighted downsampling mechanism, which represents a final global feature vector of shape 256 that, in turn, represents a low-level representation of the entire MRI. The low-level representation was then used from the classification head to produce an output that can be 0 or 1. The former value represented an intact knee, while the latter value represented an injured knee based on the injury being analyzed from the network. Additionally, to optimize the model, the weighted binary cross-entropy loss function was used. The mathematical formulation of the weighted function was as follows:

(2) $$L_{WCE}=-\alpha_{i}Y_{i}\log\left({\hat{Y}}_{i}\right)-\left(1-Y_{i}\right)\log\left(1-{\hat{Y}}_{i}\right),$$

where $\alpha_{i}$ is the weighting factor for the i-th example for 0≤i<N with N being the number of samples, $Y_{i}$ is the ground truth label for the i-th sample, and ${\hat{Y}}_{i}$ is the predicted value for the i-th sample.

**Figure 3:**
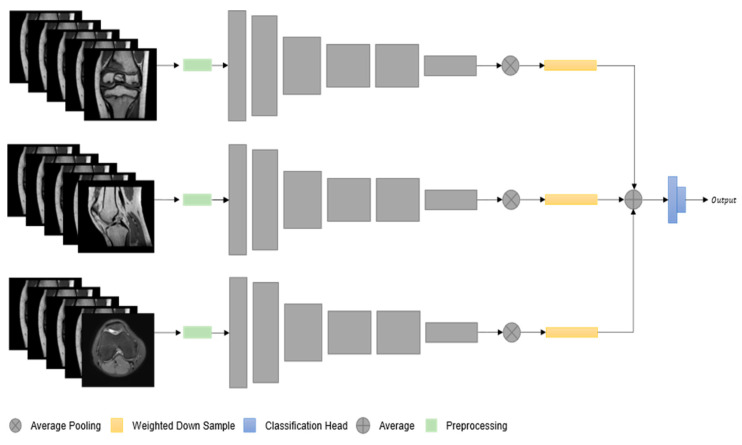
Illustration of multi-stream KNet architecture, where each stream represents a different type of MRI series that network takes as input.

The current model representrf a network that could process a single MRI slice at a time. As in real-life scenarios, radiologists usually produce multiple MRIs of different orientations and types for the knee, our KNet could be extended to simultaneously take as inputs up to three MRIs of the same patient. We named this architecture multi-stream KNet and illustrate it in Figure 3. Specifically, MS-KNet uses three separated KNet branches that are fused together through an averaging operation before moving to the classification head.

### 2.3. Dataset

Reports of knee MRI exams performed at Fisiocard Medical Center in Rome between January 2014 and October 2022 were manually reviewed to curate a dataset of knee MRI examinations. The dataset contained 401 (71.1%) abnormal exams, with 177 (31.3%) meniscus tears, (28.7%) bone edema, and 114 (20.2%) other abnormalities, such as ACL, Baker’s cyst, collateral ligaments, etc. Meniscus tears and bone edema occurred concurrently in 52 (9.2%) exams. We noted that patients with more exams were taken into consideration only when the exams belonged to different knees. Hence, at most, two exams per patient were used. Additionally, an important exclusion criterion from the dataset regarded previous interventions on the knee. Patients with previous surgical interventions (i.e., ACL reconstruction, partial meniscectomy, etc.) were excluded from the study due to the small number of cases, which is a prohibitive factor when it comes to building machine learning models. Furthermore, the interobserver rate on the evaluation of the cases by the radiologists was very low, consisting of only three cases. Those cases were categorically excluded from the study. Examinations were performed using Esaote scanners with a standard knee MRI coil and a routine non-contrast knee MRI protocol that included the following sequences: coronal hybrid (T1 and T2), sagittal T1 and T2, and axial T2 weighting for all exams. The number of image slices in each sequence ranged from 14 to 56.

For the purpose of conducting systematic experimentation in evaluating the proposed method, the dataset was further split into a training set of 466 exams (430 patients) and a test set of 98 exams (98 patients), following a random sampling approach constrained to assign a balanced number of normal and abnormal patients in the test set. In addition, all MRI types were used to train the model and conduct an ablation study to find the combination of three MRI types that gave the best result for each abnormality (meniscus tear and bone edema). More specifically, a combination of sagittal T1, sagittal T2, and coronal hybrid was used for meniscus tears detection. For bone edema, the same combination also produced better results, while for general abnormalities, a combination of axial T2, coronal hybrid, and sagittal T1 produced the best result. The overall statistics regarding the datasets are reported in Table 1.

## 3. Results

### 3.1. Evaluation Protocol

The proposed machine learning algorithm follows a paradigm named supervised learning [28]; therefore, the model needs to have a ground truth label associated with each MRI image in input. To this end, reference standard labels for each patient were obtained on the internal dataset set from the majority vote of three (musculoskeletal) MSK radiologists from the same clinic from the dataset was acquired. The MSK radiologists had access to all DICOM series, the original report and clinical history, and follow-up exams during interpretation. All readers participating in the study used a clinical picture archiving and communication system (PACS) environment, and evaluation was performed on the clinical DICOM images presented on a range of 3–12 mega pixels medical-grade display using a type Nio Fusion 12MP MDNC-12130 with a minimum luminance of 300 cd/m^2^, maximum luminance of 1200 cd/m^2^, pixel size of 0.2, and native resolution of 4200 × 2800 pixels. Exams were sorted in reverse chronological order. Each exam was assigned a binary label for the presence or absence of each of the diseases taken into consideration. Definitions for labels were as follows:

- Meniscus: intact (normal, without degenerative notes, not broken but with degenerative notes) or tear (broken, radial, longitudinal, or fracture lines present in at least three slices or morphologic deformity);
- Bone Edema: intact (normal bone, not inflamed) or inflamed (signal hyperintensity in T2 stir, inflamed bone following direct trauma or sprain);
- Abnormality: intact (if both meniscus and bone edema are intact, and no other fractures are present in the knee) or abnormal (if any fracture is present)

The learning algorithms then used these labels as ground truth target variables during the learning process, as well as to measure the performance during validation.

### 3.2. Performance Metrics

To assess the performance of our model, we decided to use three very common metrics in medical image classification problems, namely accuracy, sensitivity, and specificity [10,22]. The reason behind choosing these three metrics stands on their properties. Starting with accuracy, it is a measure of the performance of machine learning models used for classification. Its interpretation is straightforward and shows the rate of predictions performed that are correctly calculated as the number of correct predictions over the total number of predictions.

The drawback of accuracy as a measure of performance is that it is context invariant, and its interpretation is the same for every classification problem. Since, in medicine, it is very important to interpret the results, metrics such as sensitivity and specificity are commonly used. Sensitivity, also known as the true positive rate, measures the ability of a model to yield a positive result for a subject that has that disease; in other words, it can be interpreted as the probability of the model predicting that the knee has a fracture given that the knee is fractured. On the other hand, specificity, which is defined as the true negative rate, measures the ability of a model to yield a negative result for a subject that does not have a fracture, and contrary to sensitivity, it shows the probability that the model predicts a negative, given that the patient’s knee does not have any kind of fractures. Both sensitivity and specificity are very important for measuring the performance of a model and its interpretation. Usually, when there is a threshold different from 0.5 involved in separating the labels 0 and 1, one can use it to increase the specificity or sensitivity of the model based on the final goal of the application and present an inverse correlation between the specificity and sensitivity, meaning that if one is increased, the other is automatically decreased. In our case, this inverse correlation does not hold since we use a threshold of 0.5.

### 3.3. Model Performance

The model was trained on the training dataset of knee MRI scans and used to perform three distinct tasks: abnormality detection, meniscus tears detection, and bone edema detection. The model results are promising, with an accuracy of 83.7% for abnormality detection, 83.7% for meniscus tears, and 81.3% for bone edema detection. The sensitivity of the model was high for abnormality detection (90%) and bone edema detection (93.3%), while it was lower for meniscus tears detection (75%), meaning that the model is very good at identifying positive cases for the tasks of abnormality and bone edema detection. On the other hand, the specificity of the model was high for meniscus tears detection (92%), but it was lower for bone edema detection (65.2%) and abnormality detection (73.7%), meaning that the model is very good at identifying negative cases for the task of meniscus tears detection. From the results, we can notice that the model performs quite effectively for all the tasks, but there is a disbalance between sensitivity and specificity. The reason behind this phenomenon is that the disproportional distribution of negative and positive cases is also limited by the low availability of training data. However, these results indicate that the model has the potential to be a valuable tool for medical professionals in the analysis of knee MRI scans, even when trained with a small cohort of patients. The overall results are shown in Table 2.

### 3.4. Ablation Study

The evaluation of a machine learning model involves multiple performance metrics; thus, the best model is determined based on the metrics of interest. To create a better evaluation, we conducted an ablation study by testing different types of inputs. If accuracy is the primary metric, the models listed in Table 2 are the top performers, and for abnormality detection, it uses axial T2, coronal hybrid, and sagittal T1 sequences as inputs, while for meniscus tears and bone edema, the best performing model takes coronal hybrid, sagittal T1, and sagittal T2 sequences as inputs. However, if specificity or sensitivity is the focus, the results are different. In terms of specificity, the best performing model for abnormality detection only uses the axial T2 sequence as input (84.2%), while for meniscus tears, the best model remains the same, and for bone edema, the model with the highest specificity only uses the axial T2 sequence as input (78.6%). When it comes to sensitivity, the best performing models for abnormality and bone edema detection are the same as those reported in Table 2, which also produce the highest accuracy, while for meniscus tears, the model with the highest sensitivity uses the combination of Axial T2, sagittal T1, and sagittal T2 as inputs (82.8%). Lastly, if a balanced model with a small gap between accuracy, sensitivity, and specificity is desired, the best performing models may differ, and the most balanced model for all tasks is obtained from the combination of axial T2, sagittal T1, and sagittal T2 as inputs. The ablation study also checks if the model truly benefits from multiple input images or if it can perform similarly with just one sequence as input. As seen from Table 3, using multiple sequences as inputs improves the overall performance of the model in most cases; however, it is worth noting that the model still achieves good performance, even with just one input sequence.

## 4. Discussion

The objective of this study was to create and assess a machine learning model for categorizing knee MRI pathologies and compare its performance with that of clinical experts on real-life MRI imaging protocols. The findings showed that a deep learning approach could achieve a high level of accuracy in clinical classification tasks on knee MRIs, with performance accuracy rates of 83% for abnormality detection, 83.7% for meniscus tear detection, and 81.3% for BME. The model achieved high specificity in detecting meniscus tears on the internal validation set, indicating that it could be potentially effective in ruling out meniscus tears if used in the clinical workflow. However, more data may be required to improve the model’s capability for diagnosing the other two injuries. The approach also showed promise in aiding radiologists in evaluating knee MRIs, as the deep learning model can provide suggestions in a matter of seconds, in contrast to human experts, who require a significant amount of time to analyze MRIs [10]. The study’s results also suggest that deep learning represents a potential group of algorithms that can be employed to generate rapid automated pathology classifications for advanced MSK MRI. An automated deep learning model for knee MRI diagnosis has the potential to improve clinical practice in a number of ways. For example, the model could be used to prioritize diagnostic worklists by automatically moving abnormal exams ahead of normal exams in the image interpretation workflow [29]. This approach could lead to quicker preliminary feedback for patients whose exams come back as normal. Additionally, providing rapid results to the ordering clinician could improve disposition in other areas of the healthcare system [30]. In this study, radiologists reported that the use of the automated model improved their performance in detecting abnormalities in the knee in terms of time needed to identify the injury, especially for meniscus tears, given the high specificity of the results. However, for cases when the model predicted an incorrect result, the radiologist required extra amount of time to define the exact knee injury if present. This finding suggests that the model could potentially help reduce unnecessary additional testing if trained with the proper amount of data to reach higher levels of accuracy. Automated abnormality prediction could also help general radiologists or even non-radiologist clinicians (orthopedic surgeons) interpret medical imaging for patients at the point of care, rather than waiting for specialized radiologist interpretation [10]. This innovation could aid in efficient interpretation, reduce errors, and help standardize the quality of diagnoses when MSK specialist radiologists are not readily available. Our results provide early support for a future where automated models may play a significant role in assisting clinicians and healthcare systems.

One challenge in utilizing machine learning for medical imaging involves assembling large datasets that encompass a diverse array of abnormalities that can appear on a particular imaging exam, ensuring the proper training of an accurate classifier [31,32,33]. By training the model to recognize the range of what constitutes a normal exam for a specific population, it would theoretically be capable of detecting any abnormality, even exceedingly rare ones. Indeed, this need for more data to train a robust classification model represents the main limitation of this study. Our experimental cohort was composed of 564 examinations, of which 466 were used for training, with three MRI types each input into the model. Even though 466 is not a small number, in terms of learning algorithms, it is far from the optimal number. However, the fact that examinations were obtained from the same institution with evaluations performed by the same group of radiologists makes this number of exams more significant in extracting preliminary conclusions regarding the usability of such models in clinical settings. At the same time, the lack of exams from other institutions and clinicians lowers the variance in data in terms of images and evaluations, which is a limiting factor for generalizing machine learning models in terms of diagnosing knee MRI. Based on this rationale, further studies are necessary to provide bigger datasets that also enable the use of bigger and better models for medical imaging, which could mark new breakthroughs in assisted medicine.

In conclusion, this work proposes a novel machine learning model that combines the power of feature extraction algorithms to enhance the amount of information contained in each MRI sequence elements, as well as the power of machine learning models that are capable of extracting complex patterns from the data and modeling the characteristic distribution of the knee as presented in a MRI. The proposed model is applied to real-life imaging protocols and achieves high performance in terms of accuracy, sensitivity, and specificity. Even though is an early-stage study and we definitely require future works to improve the quality of the model and the quantity of the data, the provided results suggests that machine learning models can be effective potential assistants to radiologists in their day-to-day activities, helping in the diagnosis and interpretation process of knee MRIs.

## 5. Conclusions

In conclusion, this study explored the application of machine learning techniques in the identification of knee pathologies, such as medial meniscus tears and bone marrow edema, from MRI scans. The results demonstrate that machine learning algorithms have the potential to serve as valuable assistants to radiologists in evaluating MRIs, providing an efficient means of diagnosing knee pathologies. With further improvements in the algorithms, incorporation of larger datasets, and refinement of the model, machine learning-based approaches can enhance the diagnostic accuracy and efficiency in the field of knee imaging, ultimately leading to improved patient care and outcomes. Further research and collaboration between medical professionals and data scientists are warranted to optimize and validate the proposed method for routine clinical use.

## Figures and Tables

**Table 1:** Statistical data regarding used dataset.

Statistics	Training Set	Testing Set
Number of exams	466	98
Number of patients	430	98
Number of female patients	205 (46.9%)	21 (21.4%)
Mean age	46.1	39.8
Number with abnormality	335 (71.9%)	66 (67.3%)
Number with meniscus tears	127 (27.3%)	50 (51.0%)
Number with bone edema	132 (28.3%)	30 (30.6%)
Number with bone edema and meniscus tears	38 (8.2%)	14 (14.3%)

**Table 2:** Best model results for Abnormality, Meniscus tears and Bone edema.

Task	Accuracy	Sensitivity	Specificity
Abnormality	83.7%	90%	73.3%
Meniscus tears	83.7%	75%	92%
Bone edema	81.3%	93.3%	65.2%

**Table 3:** Ablation study on different input combinations to model.

	Abnormality			Meniscus Tears			Bone Edema		
Input	Acc.	Sens.	Spec.	Acc.	Sens.	Spec.	Acc.	Sens.	Spec.
Coronal hybrid	79.5%	85.2%	73.9%	73.4%	62.5%	80.0%	79.5%	85.2%	73.9%
Axial T2	75.5%	66.8%	84.2%	79.5%	70.8%	83.7%	75.5%	71.4%	78.6%
Sagittal T1	77.6%	76.9%	78.7%	77.6%	68.2%	85.5%	77.6%	80.8%	73.9%
Sagittal T2	77.6%	76.9%	78.7%	75.5%	67.5%	83.7%	77.6%	80.8%	73.9%
Coronal hybrid and sagittal (T1 and T2)	81.6%	86.7%	73.7%	83.7%	75.5%	92.0%	81.6%	93.3%	65.2%
Axial T2, coronal hybrid, and sagittal T1	83.7%	90%	73.7%	79.9%	70.5%	87.99%	79.9%	85.2%	73.9%
Axial T2 and Sagittal (T1 and T2)	79.5%	80.3%	78.7%	81.7%	82.8%	80.8%	77.6%	78.3%	75.6%

## Data Availability

Data are contained within the article.
